# The Variable Effect of Polyploidization on the Phenotype in *Escallonia*

**DOI:** 10.3389/fpls.2018.00354

**Published:** 2018-03-20

**Authors:** Hanne E. R. Denaeghel, Katrijn Van Laere, Leen Leus, Peter Lootens, Johan Van Huylenbroeck, Marie-Christine Van Labeke

**Affiliations:** ^1^Applied Genetics and Breeding, Plant Sciences Unit, Flanders Research Institute for Agriculture, Fisheries and Food, Melle, Belgium; ^2^Department of Plant Production, Faculty of Bioscience Engineering, Ghent University, Ghent, Belgium

**Keywords:** chromosome doubling, cold tolerance, compactness, image analysis, plant architecture, rooting capacity

## Abstract

To induce new variation within the *Escallonia* genus, chromosome doubling was performed in *E. rubra, E. rosea*, and *E. illinita*, three important species within this genus of mainly evergreen woody ornamental species. Obtained tetraploids and diploid controls were analyzed for rooting capacity, leaf and flower characteristics, and plant architecture using image analysis and cold tolerance. In the present study, a breeders' collection of 23 accessions was characterized cytogenetically and described morphologically. All analyzed species and cultivars were diploid (2n = 2x = 24), with exception of *E. pendula*, a tetraploid. Today, breeding in *Escallonia* is limited to lucky finds in seedling populations and few efforts in interspecific hybridization. Three selected *Escallonia* species underwent an *in vitro* chromosome doubling with both oryzalin and trifluralin applied as either a continuous or shock treatment. The treatments successfully induced polyploids in all three species. Image analysis revealed that tetraploid *E. rosea* had decreased shoot length (from 3.8 to 1.3 cm), higher circularity and more dense growth habit compared to diploids. No significant changes in cold tolerance were seen. Tetraploid *E. illinita* did not differ in shoot length, but an increased outgrowth of axillary buds on the main axis led to denser plants. For tetraploid *E. rubra*, an increase in plant height (from 4.9 to 5.5 cm) was observed together with a large decrease in circularity and density due to a more polar outgrowth of branches on the main axis. *E. rubra* tetraploids bore larger flowers than diploids and had an increased cold tolerance (from −7.7 to −11.8°C). Leaf width and area of tetraploids increased for both *E. illinita* and *E. rubra*, while a decrease was seen in *E. rosea* genotypes. For all three species, the rooting capacity of the tetraploids did not differ from the diploids. We conclude that the effect of polyploidization on *Escallonia* was highly variable and species dependent.

## Introduction

Polyploidization is a breeding tool that creates variation in phenotype and physiology (Horn, 2002; Dhooghe et al., 2011; Sattler et al., 2016). The basic consequence of polyploidization is an increase in cell size caused by the larger number of gene copies (gigas effect) (Sattler et al., 2016). Therefore, polyploids may have larger organs than their diploid counterparts, such as larger and thicker leaves, flowers, and fruits (Tang et al., 2010; Feng et al., 2017). However, increased cell size does not implicate increased plant size as the number of cell divisions can be reduced in polyploids and thus result in more compact growing genotypes (Horn, 2002; Sattler et al., 2016; Hias et al., 2017). Development of compact growing plants is a major goal in many breeding programs. Currently, compactness is obtained by frequent pruning (Meijon et al., 2009; Mutlu and Kurtulan, 2015), by changing environmental factors such as light and temperature regimes for indoor plants (Clifford et al., 2004; Löfkvist, 2010; Bergstrand and Schussler, 2013), or by using plant growth regulators such as chlormequat, paclobutrazol, trinexapac-ethyl, and daminozide (Meijon et al., 2009; Löfkvist, 2010; Lutken et al., 2012; Mutlu and Kurtulan, 2015; PPDB, 2017). The chemical approach is currently under debate for environmental reasons (Wang et al., 2011; Lutken et al., 2012). More compact growing polyploids have been found in *Malus* (Hias et al., 2017), *Buddleja* (Rose et al., 2000), *Petunia* (Regalado et al., 2017), *Rosa* (Feng et al., 2017), *Platanus* (Liu et al., 2007), and *Eriobotrya* (Blasco et al., 2015) among others. Besides morphological changes, physiological changes, e.g., stress resistance and flowering period, are reported for polyploid plants (Levin, 2002; Van Laere et al., 2010; Regalado et al., 2017) due to an increase in genome flexibility (Levin, 2002). A change in drought tolerance was found in tetraploid *Spathiphyllum* (Van Laere et al., 2010), in autotetraploid *Malus* (Zhang et al., 2015) and in pentaploid *Betula* (Li et al., 1996). An increase in drought tolerance was found in *Citrus* (Ruiz et al., 2016). In tetraploid *Lonicera*, an increase in both heat tolerance and drought was observed (Li et al., 2009, 2011).

*In vivo* and *in vitro* polyploidy induction has been widely applied in ornamental breeding. However, each species responds differently to polyploidization and tailored protocols need to be developed. Most protocols are developed *in vitro*, but some species are very difficult to initiate and propagate *in vitro*, and *in vivo* chromosome doubling protocols are needed, e.g., for *Ziziphus* (Shi et al., 2015). Once polyploids are created, traits need to be evaluated. Most phenotyping studies on polyploid ornamentals measure the characteristics of interest, e.g., internode length, leaf length and width, flower diameter, number, and length of branches, etc., manually on a limited number of plants (Stanys et al., 2006; Liu et al., 2007; Tang et al., 2010; Van Laere et al., 2010; Regalado et al., 2017). However, in contrast, image analysis enables a more in-depth analysis of plant characteristics (Fahlgren et al., 2015), as performed on leaf area on tetraploid apple trees (Hias et al., 2017). Image analysis of the whole shoot augmented the information and the number of plants that could be analyzed in rose (Li-Marchetti et al., 2015) and pea (Humplik et al., 2015a).

The present study was conducted on the genus *Escallonia* Mutis ex L.f. This genus belongs to the Escalloniaceae family and contains about 40 flowering woody ornamental species, endemic to South America, especially Chile. Most species are evergreen, have white to red flowers, a honey fragrance, and resin glands on leaves and branches (Bean and Murray, 1989). *Escallonia* is used as a hedging plant, especially in coastal regions. Depending on the species, *Escallonia* can thrive in USDA zones from 10 to 7b, which corresponds to mean minimum temperatures ranging from −1.1 to −14.9°C (Hoffman and Ravesloot, 1998). Numerous hybrids and cultivars have been described, and many of them have an *E. rubra* and *E. virgata* background, such as the F1 hybrids *E*. “Langleyensis” and *E*. “Edinburgh” (= *E*. “Edinensis”). *E*. “Donard Seedling,” *E*. “Apple Blossom,” and *E*. “Slieve Donard” are backcrosses of *E. virgata* × *E*. “Langleyensis,” while *E. rubra* “C.F. Ball” is a backcross of *E. rubra* var. *macrantha* × (*E. rubra* × *E. rubra* var. *macrantha*). The genotype *E*. “Red Elf” is an *E. rubra* “C.F. Ball” and *E*. “William Watson” hybrid, with the latter being a seedling of *E*. “Langleyensis” (Krüsmann, 1960; Bean and Murray, 1989; Hilliers Garden, 1991). Genetic and cytogenetic data of *Escallonia* are rarely available. Chromosome counts were performed for 21 *Escallonia* genotypes; all are reported to be diploid with 24 chromosomes (Darlington and Wylie, 1955; Zielinski, 1955; Hanson and Leitch, 2002).

The aims of this study were (1) cytogenetic analysis of a breeders' collection of Escallonia; (2) induction of polyploids of a selected number of *Escallonia* species; and (3) quantification of horticultural traits, plant architecture, winter hardiness and rooting capacity in generated tetraploids.

## Materials and methods

### Plant material

Our collection contained 23 *Escallonia* accessions. Table 1 presents the acquisition origin, reported winter hardiness and some morphological characteristics such as height and flower color. All genotypes were acquired in 3-fold, one plant was planted in the field (51°0′N, 3°48′E, Melle, Belgium) and two were grown as a container plant in a peat-based substrate (Saniflor: 1.5 kg/m fertilizer: 12N:14P:24K + trace elements, pH 5.0-6.5, EC 0.450 mS/cm) and kept in a frost-free glasshouse (min. temperature 6°C).

**Table 1:** The collection of *Escallonia* genotypes with their acquisition origin and accession number and observed morphologic and cytogenetic characteristics.

***Escallonia* genotype**	**Acquisition**		**Winter hardiness (°C)^b^**	**Flower color**^**c**^		**Plant height (m)^c^**	**2C-value (pg)**	**Chromosome number (*n* = 12)**
	**Grower/botanical garden^a^**	**Accession number**		**Literature**	**Own observation**			
*E. alpina* DC. (1)	HG		−6.6	Red	White	0.6–3.5	1.16 ± 0.07	24
*E. alpina* DC. (2)	BRMB			Red	=		1.13 ± 0.04	24
*E*. ‘Apple Blossom’	PE		−14.9	White with pale rose	=	1.5–2.5	1.13 ± 0.03	24
*E. bifida* Link & Otto	BGM	19084138	−6.6	White	=	>3	1.43 ± 0.03	24
*E*. ‘Donard Seedling’	DN		−14.9	White with pale rose	=	≥2.5	1.07 ± 0.06	24
*E*. ‘Edinburgh’	RBGE	20050334 A	−12.2	Rosy carmine	=	–	–	24
*E. illinita* Presl. (1)	DN		−12.2	White	White with pale rose	3	1.18 ± 0.04	24
*E. illinita* Presl. (2)	BGUW	XX-0-WU-ESC000001		White	=		1.31 ± 0.08	24
*E*. ‘Iveyi’ (Veith)	DN		−14.9	White	=	>2.5	1.32 ± 0.16	24
*E. iveyi* (x) Veith	BGM	19391832		White	=		1.25 ± 0.03	24
*E. laevis* ‘Gold Ellen’	DN		−12.2	Dark rose	=	–	1.21 ± 0.12	24
*E*. ‘Langleyensis’ (Veith)	TS	1347	−12.2	Rosy carmine	=	2.5	1.06 ± 0.02	24
*E. myrtoidea* Bertero ex DC.	RBGE	20130304	–	–	–	–	1.20 ± 0.03	24
*E. pendula* Ruiz & Pav.	BGM	19843175	–	White	–	>3	2.17 ± 0.16	48
*E*. ‘Red Dream’	DN		−12.2	Red	=	–	1.20 ± 0.12	24
*E*. ‘Red Elf’	DN		−12.2	Red	=	1.5–2.5	1.11 ± 0.06	24
*E. rosea* Griseb.	HG		–	White	White with pale rose	1–2.5	1.11 ± 0.06	24
*E. rubra* (Ruiz & Pav.) Pers.	RBGE	19924317*B	−12.2	Rose to deep crimson	=	4.5	1.07 ± 0.06	24
E. *rubra* ‘C.F. Ball’	BGM	19810040		Crimson	=	2.1–3	1.07 ± 0.03	24
*E. rubra* var. *macrantha* (Hook. & Arn.) Reiche	DN			rose to red	=	1.8–3	1.12 ± 0.03	24
E. *rubra* var. *rubra*	HB	C0834		–	Red	–	1.12 ± 0.02	24
*E*. ‘Slieve Donard’	RBGE	19653608 A	−14.9	Lightly rose with carmine	=	1.5–2.1	–	24
*E. stricta* (x) Remy.	HG		–	Red	=	–	1.15 ± 0.04	24

*^b^Winter hardiness, ^c^flower color and ^c^plant height derived from literature were compared to the acquired plants. 2C value and chromosome number were measured*.

*^a^DN, tree nursery De Neve, Oosterzele, Belgium; BGM, Botanical Garden Meise, Meise, Belgium; RBGE, Royal Botanical Garden Edinburgh, Edinburgh, Scotland; HG, Hillier Gardens, Ampfield, Romsey, UK; BGUW, Botanischer Garten Universität Wien, Vienna, Austria; HB, Hortus Botanicus, Amsterdam, The Netherlands; BRMB, Boomkwekerij Rein & Mark Bulk, Boskoop, The Netherlands; TS, Ter Saksen, Beveren, Belgium; PE, Plantentuin Esveld, Boskoop, The Netherlands*.

*^b^Hoffman and Ravesloot, 1998*.

*^c^Data from Bean and Murray (1989), Hilliers Garden (1991), and Krüsmann (1960). −, no information available. Own observation, when matching literature; −, is noted, when no flowers appeared; −, is noted. Otherwise, the observed color is described*.

Three species (*E. illinita* Presl. (1), *E. rosea* Griseb. and *E. rubra* (Ruiz & Pav.) Pers.) were chosen for further experiments. They displayed differences in growth rate, size and plant habitus, as observed in the collection and verified in literature (Table 1).

Young shoots of the greenhouse grown plants were sterilized and nodes were initiated on a Murashige and Skoog (MS) medium (Murashige and Skoog, 1962) (MS including vitamins, Duchefa Farma, Haarlem, The Netherlands) containing 30 g/L sucrose, 2 mg/L 6-benzylaminopurine (BAP), 0.1 mg/L 1-naphtaleneacetic acid (NAA), 7 g/L agar (Lab M Limited, Heywood, Lancashire, UK) with pH = 5.9 ± 0.1. Shoots that developed on the nodes were transferred after 2–10 weeks to MS growth medium (Duchefa) containing 30 g/L sucrose, 0.15 mg/L BAP, 0.05 mg/L NAA, 7 g/L agar (Lab M), and pH = 5.9 ± 0.1. They were grown in the growth chamber (ambient temperature: 23 ± 1°C, photoperiod: 16 h, light intensity: 35 μmol m^−2^s^−1^, bottom cooling: 18 ± 1°C) in a 500 mL jar. The *in vitro* stock, which was started 6–12 months before the experiments, was sub-cultured on fresh medium every 3–4 months.

### AFLP analysis

AFLP was performed on the *Escallonia* collection. For DNA extraction, the modified CTAB DNA isolation protocol of Doyle and Doyle (1987) was used with 20 mg of lyophilized young leaf material as starting material. AFLP reactions were executed according to the protocol described by Van Laere et al. (2011a). AFLP reactions were run on an ABI Prism 3130xl Genetic Analyzer (Applied Biosystems, Foster City, CA, USA) with the GeneScan 500Rox kit for labeling (Applied Biosystems). Four fluorescent-labeled EcoRI and MseI primer combinations with six selective bases were used for the selective amplification, i.e., E-AAC + M-CTG, E-ACA + M-CAT, E-ACT + M-CTA and E-AGG + M-CTT. Peaks lower than 50 were removed from the analysis, together with all markers occurring in either more than 95% or <5% of the population. A phylogenetic tree was constructed using a UPGMA clustering method with Jaccard indices and bootstrap values, executed in R (R version 3.2.0) (R Core Team, 2015).

### Chromosome counts and determination of 2C value

All genotypes in the collection were analyzed for 2C value and chromosome number. The 2C values were determined with a flow cytometer equipped with a laser (488 nm, 20 mW solid state laser, Sapphire 488-20) (Pas III, Partec). Samples were chopped according to Galbraith et al. (1983). Staining with propidium iodide (PI) was performed using the commercially available PI Cystain kit (Partec). The samples were incubated in the dark at 5°C for at least 30 min before measuring. As internal standard the tomato *Solanum esculentum* “Stupicke” (2C = 1.96 pg) (Dolezel et al., 1992) was used. Each measurement was repeated at least five times on at least 3 days. Histograms were analyzed using FloMax software (Partec). Terminology on 2C values was used as defined by Greilhuber et al. (2005). The ratio between the peaks of tomato and the *Escallonia* genotype measured was multiplied with the 2C value of tomato to determine the *Escallonia* genotype.

Chromosome numbers from the plants in the collection were determined from actively growing root meristems. Young root tips (±1 cm) were incubated for 3 h in an antimitotic mixture (2.5 mM colchicine + 1 mM 8-hydroxyquinoline) and fixated in 3:1 EtOH:acetic acid for 45–60 min at room temperature. Digestion was performed at 37°C for 60–100 min (depending on the genotype) using 1% enzyme suspension (1% cellulase and 1% pectolyase in 10 mM citric acid buffer). Slides were made according to the SteamDrop protocol (Kirov et al., 2014) with 2:1 and 1:1 EtOH:acetic acid as the first and second fixative, and stained with 4′,6-diaminidino-2-phenylindole (DAPI, 1 mg/mL). Slides were examined using fluorescence microscopy (Leica DMIRB, 1000x, LEICA microsystems) equipped with a DFC450 camera and LAS software (LEICA microsystems). Chromosome analyses were carried out on 10 well-spread metaphases of each genotype by DRAWID software version 0.26 (http://drawid.xyz/; Kirov et al., 2017).

### Chromosome doubling

#### Shock treatments

Nodal explants (0.2–0.5 mm) of *E. illinita, E. rosea*, and *E. rubra* were submerged in 100 mL liquid MS medium (Duchefa) with 30 g/L sucrose, pH = 5.9 ± 0.1, with addition of 0, 50, 150, or 250 μM of oryzalin (ORY; dissolved in 1 mL 99% EtOH) or trifluralin (TRI; dissolved in 1 mL acetone). The nodes were incubated for 2, 3, or 4 days on a gyratory shaker (60 rpm) in a growth chamber (conditions as above). Each treatment was performed on 30 nodes (2 jars with 15 nodes). The control treatment (0 μM; only addition of 1 mL EtOH or acetone in analogy with the treatments) contained 18 nodal explants (2 jars with 9 nodes). When the allotted exposure time was reached (2, 3, or 4 days) the nodal explants were rinsed with sterile demineralized water and transferred to solid MS growth medium (Duchefa), with 30 g/L sucrose, 0.15 mg/L BAP, 0.05 mg/L NAA, 7 g/L agar (Lab M), and pH = 5.9 ± 0.1, (6 nodes per jar) and placed in the growth chamber.

#### Continuous treatments

Nodal explants (0.2–0.5 mm) of *E. illinita, E. rosea*, and *E. rubra* were placed on 100 mL solid MS growth medium containing either 0, 1, 5, or 10 μM ORY (dissolved in 40 μL EtOH) or TRI (dissolved in 40 μL acetone) and grown in the growth chamber (for conditions see above) for 6, 8, or 10 weeks. Each treatment was performed on 30 nodes (6 per jar). The control treatment (0 μM; only addition of 40 μL EtOH or acetone in analogy with the treatments) contained 18 nodal explants (6 per jar). After the allotted exposure time (6, 8, or 10 weeks), the nodal explants were rinsed with sterile distilled water, transferred to 100 ml solid MS growth medium (Duchefa), with 30 g/L sucrose, 0.15 mg/L BAP, 0.05 mg/L NAA, 7 g/L agar (Lab M), and pH = 5.9 ± 0.1, (6 nodes per jar) and placed in the growth chamber.

#### Analysis of ploidy level with flow cytometry

The mortality (%) and tetraploid yield (T-yield, %) of the shock and continuous treatments were determined 12 weeks after the start of the experiment. From each surviving nodal explant, a single developing axillary shoot was selected, indicating one possible polyploidization event. To determine the ploidy level, a young leaf was sampled (10 à 20 mm^2^). Samples were chopped according to Galbraith et al. (1983), stained with 4′,6-diaminidino-2-phenylindole (DAPI) using a citrate buffer (500 μL 0.1 M citric acid monohydrate and 0.5% Tween 20) and a phosphate buffer (750 μL 0.4 M Na_2_HPO_4_.12H_2_O, 2 mg/L DAPI, 0.1% polyvinylpyrrolidone (PVP) (modified from Otto, 1990), and analyzed with a flow cytometer equipped with a UV LED (365 nm) (Cyflow Space, Partec). Histograms were analyzed using FloMax software (Partec). An *in vitro* sample of a non-treated diploid plant of the same species was used to calibrate the flow cytometer.

After acclimatization in the greenhouse, 10 diploid controls and 23 tetraploid numbers of *E. illinita* were randomly chosen from all acclimatized plantlets for further phenotyping. For *E. rosea* and *E. rubra*, 6 and 23 tetraploid numbers, respectively, and 4 and 16 diploid controls, respectively, were obtained from a similar, preliminary experiment with ORY and TRI (Denaeghel et al., 2015). Each event that resulted in a tetraploid was identified by a unique code (T01, T02,…) for each species. Diploid plants from the control treatment were similarly named (D01, D02,…). The diploid controls and tetraploid events are therefore referred to by these codes below.

### Characterization of the tetraploids

#### Plant material

All tetraploid shoots and an equal number of diploid shoots from the control treatment were transferred to a solid MS (Duchefa) rooting medium (30 g/l sucrose, 0.5 mg/l NAA, 7 g/l agar (Lab M), pH = 5.9 ± 0.1) for approximately 6 weeks. When the first roots emerged, plantlets were acclimated in the greenhouse (16L:8D; mean temperature day: 22.9°C and night: 19.9°C; fertilization: N-P-K+MgO 20-5-10+2 at EC = 1 mS/cm and pH = 5) in a peat based substrate (Saniflor: 1.5 kg/m^3^ fertilizer: 12N:14P:24K + trace elements, pH 5.0-6.5, EC 0.450 mS/cm). These plants were used as mother plants for cutting production. Cuttings were rooted in peat substrate (Saniflor: 1.5 kg/m^2^ fertilizer, 12N:14P:24K + trace elements, pH 5.0-6.5, EC 0.450 mS/cm) without auxin treatment in the greenhouse under a tunnel covered with white plastic to maintain humidity at 100% RH. Rooted cuttings were used for further experiments.

#### Rooting capacity

For this experiment, the diploid and tetraploid numbers were grouped in homoploid groups. For *E. rosea* and *E. rubra*, 4 replicates of ±50 cuttings (±5 cm) were made from the diploid control group and from the tetraploid group. For *E. illinita*, 2 replicates of ±60 cuttings (±5 cm) were made per group. The cuttings were taken from randomly chosen numbers in the homoploid groups. After 5 weeks, the rooted cuttings were rinsed with tap water, photographed, the roots were excised and the fresh weight of the roots was determined on an analytical balance. Subsequently, the roots were dried for 48 h at 70°C and weighed again to determine the dry matter content.

#### Morphological traits

##### Growth, branching, and leaf characteristics

Apical cuttings (±5 cm) were made for each number from 6-month-old mother plants. Five weeks after cutting, measurements were made on at least four rooted cuttings per number. The length of the new apical shoot (NSL) and its internode length (NSIL) were determined. Subsequently, the cuttings were pinched, leaving four nodes of the new grown shoot. Seven weeks after pinching, the axillary budburst (% of nodes on the main stem that sprouted), axillary branch length (BL) and the branch internode length (BIL) were measured on the plantlets. Ten full-grown leaves were collected on each of five randomly chosen plantlets of each tetraploid and diploid number. Leaf length, width, and surface area were measured in ImageJ. The length was measured from the leaf base to the tip. The width was measured on the widest point of the leaf, perpendicular to the height.

##### Flower characteristics

Tetraploid and diploid mother plants were planted in the field (51°0′N, 3°48′E, Melle, Belgium) in October 2016 for *E. rosea* and *E. rubra* and in October 2017 for *E. illinita*. At the time of this writing, flowering has occurred only on *E. rubra* diploids and tetraploids and on *E. rosea* diploids. The start and end date of flowering were recorded. The flowers of the diploid controls and tetraploid genotypes were grouped. For the diploid group, 96 flowers were collected vs. 93 flowers for the tetraploid group. In addition, 76 flowers were collected from the original plant in our breeders' collection in the field. Top and side view photographs were taken of the flowers then analyzed for flower length, tube width and corolla surface area using ImageJ (Abramoff et al., 2004). The flower length was measured from the base of the receptacle to the corolla.

#### Plant architecture

**Figure 1:**
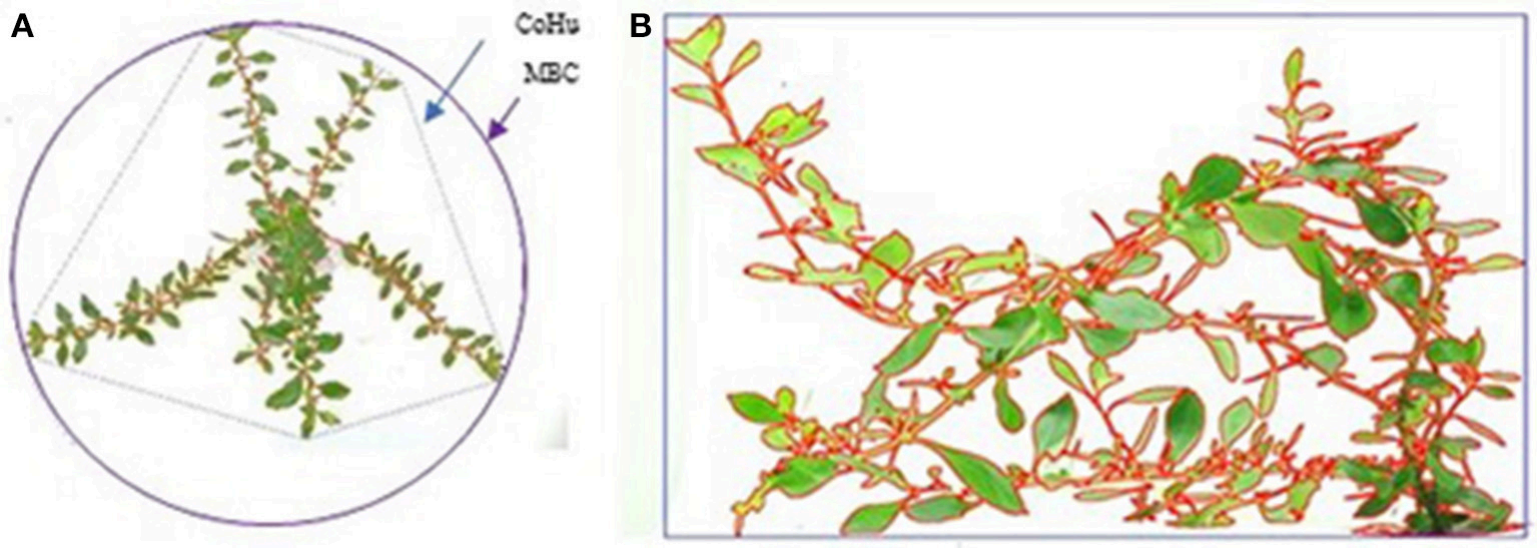
**(A)** Side view photograph of the tetraploid *Escallonia rubra* T04 with the bounding rectangle (blue) and analyzed plant area (red) indicated. **(B)** Top view photograph of the diploid *E. rubra* D04 with the convex hull (CoHu, blue), the minimal bounding circle (MBC, purple), and the analyzed plant area (red) indicated.

Eleven weeks after pinching the plantlets, photographs were taken of the individual *E. rosea* and *E. rubra* diploid and tetraploid number. From each tetraploid number and each diploid control, 10 clones were photographed overhead. For five clones, three side views were taken after turning the plant 120° after each photograph. For the top view, convex hull (CoHu) area and perimeter, and the minimal bounding circle (MBC) were determined in ImageJ (Abramoff et al., 2004; Figure 1A). The convex hull was created with the Hull and Circle plugin provided by ImageJ (Abramoff et al., 2004). With the convex hull area and perimeter, the circularity (Circ) was determined. This formula returns a value between 0 and 1, with 1 being a perfect circle.

(1) $$\begin{array}{l} Circ=\frac{4\;*\;\pi \;*\;CoHu\;area}{{\left(CoHu\;perimeter\right)}^{2}} \end{array}$$

For the side view, the plant surface, width and height were determined in ImageJ (Abramoff et al., 2004; Figure 1B), by using the Measure function (bounding rectangle) on the selected plant surface.

#### Controlled freezing test

Shoots of at least one diploid and one tetraploid number of *E. rosea* and *E. rubra* were collected after a cold period (13 days in November and December 2016 with minimum temperatures <0°C) on field-grown plants. The analyzed numbers were chosen randomly. No cold tolerance test could be performed on *E. illinita*, as they had not yet been cold-acclimated. The shoots were dissected in stem pieces of ±1 cm, each containing one axillary bud. Ten randomly chosen stem pieces were weighed on an analytical balance, dried for 48 h at 70°C, then weighed again to determine the dry matter content. Fifty stem pieces per number were placed in Eppendorf tubes (2 mL) with 0.5 mL distilled water and a few clean grains of sand. The stem tissue was frozen in a cryostat (Polystat 37, Fisher Scientific, Waltham, MA, USA) from 0 to −35°C at a rate of 6°C h^−1^. This was done for five replicates per cultivar and per freezing temperature. The positive controls were kept at a reference temperature of 4°C, the negative controls at −80°C. After the target temperatures were reached, the samples were transferred to vials containing 10 mL of incubation medium with 0.002% Triton-X and 10 mM boric acid (Ögren et al., 1997). The vials were shaken (200 rpm) for 20 h. To determine the degree of injury, the electrical conductivity (EC) of the samples was determined before (EC_samp_) and after autoclaving (EC_samp,aut_). The positive control was used as reference (EC_ref_ and EC_ref,aut_). The index of injury (Flint et al., 1967) was determined for each sample as follows:

(2) $$\begin{array}{l} I\left(t\right)=\frac{\left(\frac{\text{EC samp}}{\text{EC samp},\text{aut}}-\frac{\text{EC ref}}{\text{EC ref},\text{aut}}\right)}{1-\frac{\text{EC ref}}{\text{EC ref},\text{aut}}}\;*\;100 \end{array}$$

The formula to determine the index of injury [I(t)] is based on the principle that when dying plant cells burst, they release their content, and thus the EC value of the surrounding solution rises, which was measured after the cold treatment and the shaking. Subsequently, the surviving cells were killed by autoclaving and the total EC value was determined. The EC values of the reference samples at 4°C take the damage caused by sampling and the experiment itself into account. To calculate the LT50, the % of injury at −80°C was interpreted as 100%, and the other injury values were compared with this value. LT50-values (the temperature where 50% of plant cells were injured) were determined via sigmoidal regression on the adjusted injury values as determined by Lim and Arora (1998).

### Statistical analysis

Statistical analysis was done in R, version 3.2.0 (R Core Team, 2015). All data were first analyzed for normality using the Shapiro-Wilk Test (*p* = 0.05). For data that were not normally distributed, group comparison was done with the Kruskal-Wallis Test (KWT) and pairwise comparison with the Mann-Whitney *U*-Test (MWUT). This was done for the data on rooting capacity and the data on root dry weight. MWUT and KWT were also used for part of the data concerning growth and branching, namely BL, BIL, and axillary budburst of *E. illinita*, all the characteristics of *E. rosea*, and the BL, BIL, and axillary budburst of *E. rubra*. The data of the leaf sizes of *E. rosea* and *E. rubra* were not normally distributed, as were the data from the top view and side view images of *E. rosea* and *E. rubra*, and the length and width of the flower tube of *E. rubra*. All data were analyzed using *p* = 0.05 unless stated otherwise. Boxplots for the figures in Supplementary Data were plotted in R, version 3.2.0 (R Core Team, 2015) using the boxplot function. The bottom and top of the box are the lower and upper quartiles; the band in the middle displays the median. The upper/lower whisker extends to the highest/lowest value, up to a maximum length of 1.5 times the box length. Higher/lower values are indicated as a dot. The diploid and tetraploid numbers were sorted by means.

For normally distributed data, group comparisons were performed with ANOVA and pairwise comparison was done using the *t*-test. This was done for the data on growth and branching, namely NSL and NSIL of *E. illinita*, and the NSL and NSIL of *E. rubra*. The data of the leaves of *E. illinita* and the area of the flower top view were analyzed using ANOVA and the *t*-test.

The phylogenetic trees were plotted with the pvclust package, using the UPGMA clustering method with Jaccard indices in R (version 3.2.0) (R Core Team, 2015).

A principal component analysis (PCA) was executed for the diploids and tetraploids of *E. illinita, E. rosea*, and *E. rubra*. All three genotypes were analyzed for obtained morphological traits, namely the length of the new apical shoot (NSL) and its internode length (NSIL), the axillary budburst (BB), axillary branch length (BL) and the branch internode length (BIL). PCA of *E. rosea* and *E. rubra* included the plant architecture traits from the pictures in top view (TV), namely the plant area (TV_pl_ar), the circularity (TV_circ) and the % of the area of the minimal bounding circle that was filled by the plant (TV_fill). The architecture traits from the pictures in side view (SV) used were the plant area (SV_pl_ar), the plant height and width (SV_he and SV_wi, respectively) and the % of the area of the bounding rectangle that was filled by the plant (SV_fill). The graphs were made in R (version 3.2.0) using the prcomp function and plotted with the ggbiplot function, with ellipses drawn with a probability of 0.95 (R Core Team, 2015).

## Results

### Characterization of the collection

**Figure 2:**
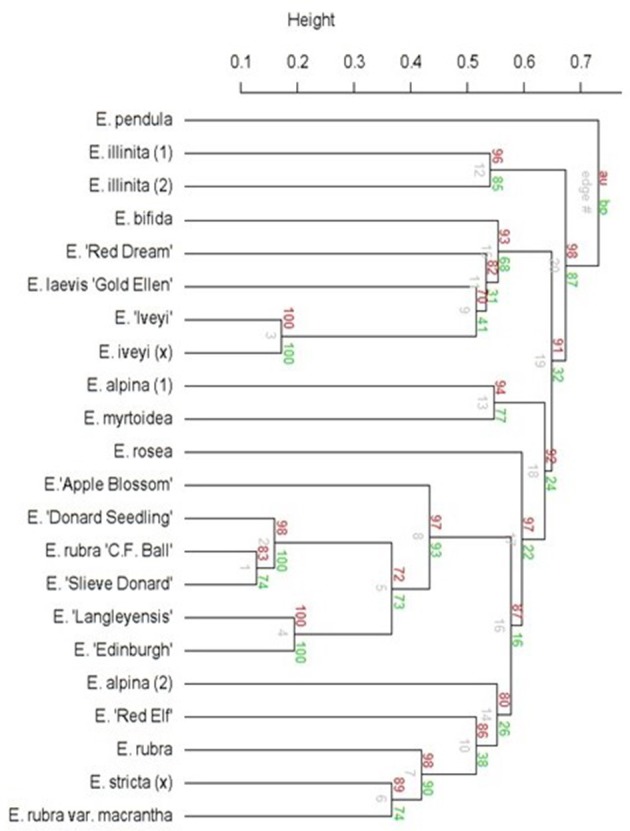
Phylogenetic tree of the collection of *Escallonia* species. AU, Approximately Unbiased *p*-values, computed by multiscale bootstrap resampling (red). BP, Bootstrap Probability value (green).

In the collection, 22 genotypes were diploid with 24 chromosomes. *E. pendula* was a tetraploid with 48 chromosomes (Table 1). Genome sizes ranged from 1.06 ± 0.02 pg/2C to 1.43 ± 0.03 pg/2C for the diploid genotypes. The tetraploid *E. pendula* had a genome size of 2.17 ± 0.16 pg/2C. Based on a brief morphological characterization and comparison of the genotypes in our collection with descriptions in botanical guides (Krüsmann, 1960; Bean and Murray, 1989; Hilliers Garden, 1991), we observed differences in flower color between the two genotypes of *E. alpina* and of *E. illinita*. The flower color of *E. alpina* (2) and *E. illinita* (2) were as described in literature (Krüsmann, 1960; Bean and Murray, 1989; Hilliers Garden, 1991). *E. alpina* (1) and *E. illinita* (1) had a deviating flower color. The flower color of *E. rosea* did not match the descriptions (Krüsmann, 1960; Bean and Murray, 1989; Hilliers Garden, 1991). The phylogenetic tree resulting from the AFLP analysis (Figure 2) shows that the two *E. illinita* genotypes and the two *E. alpina* genotypes differed. *E. pendula* was most distant related to the other *Escallonia* genotypes.

*E. illinita* (1), *E. rosea*, and *E. rubra* were chosen for further experiments. *E. illinita* (1) is a tall (2–3 m) shrub with a loose plant habitus comprised of long, arching branches. *E. rosea* is a smaller (1–1.5 m), densely branched shrub. *E. rubra* is the tallest of the three species (up to 4.5 m), but densely branched and slower growing than *E. illinita* (1). Also based on AFLP data, these three species did not show a close relation (Figure 2).

### Chromosome doubling

#### Shock treatments

**Figure 3:**
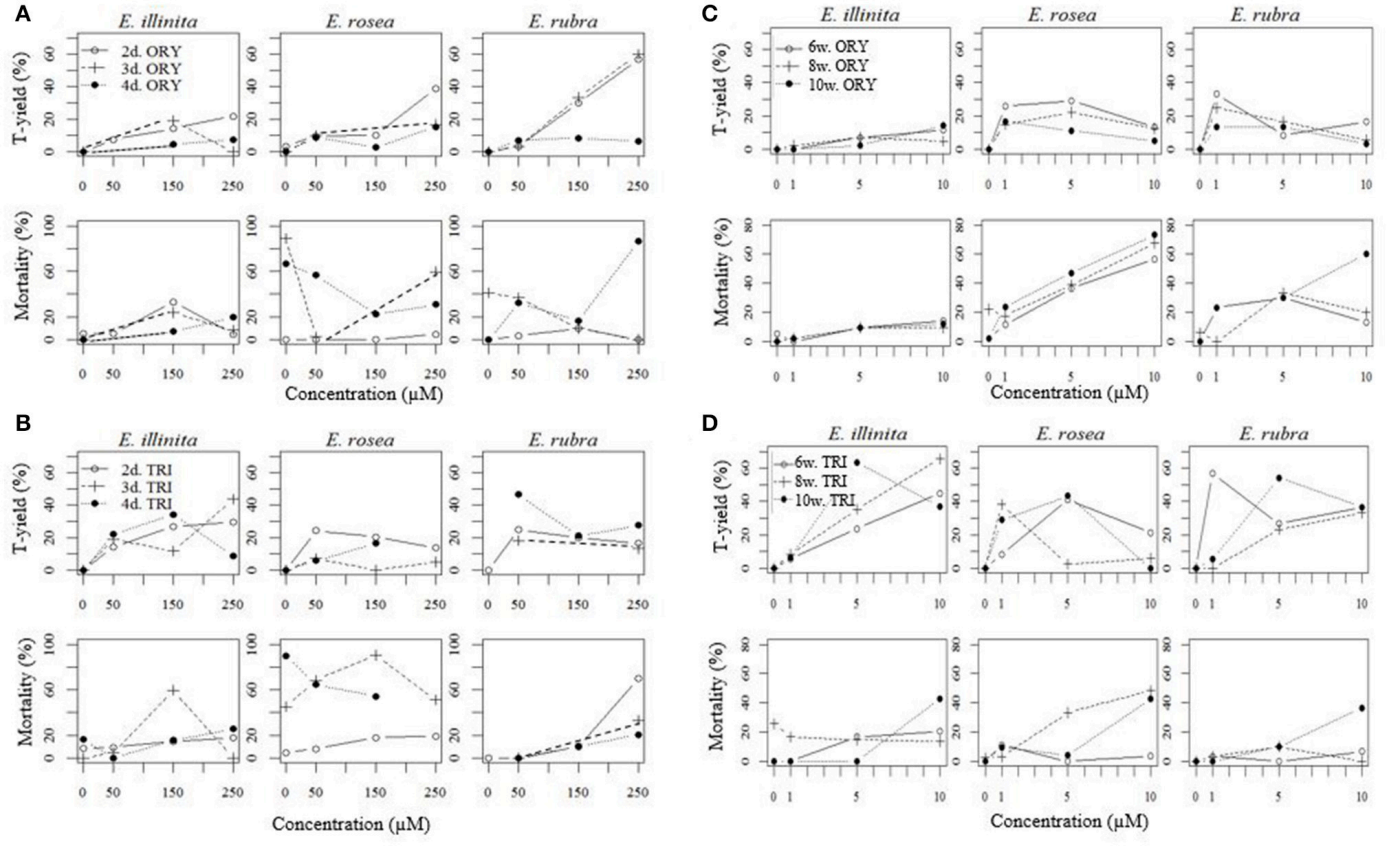
Tetraploid yield (T-yield, %) and mortality for *Escallonia illinita, E. rosea*, and *E. rubra* in a shock experiment of 2, 3, and 4 days with 0 (control), 50, 150, and 250 μM of **(A)** oryzalin (ORY) or **(B)** trifluralin (TRI), and in a continuous experiment of 6, 8, and 10 weeks with 0 (control), 1, 5, and 10 μM of oryzalin (ORY) **(C)** and trifluralin (TRI) **(D)**. Graphs plotted in R (version 3.2.0) (R Core Team, 2015).

The tetraploid yield (T-yield, %) and the mortality (%) of the shock treatments are shown in Figures 3A,B. In *E. illinita*, on average a higher T-yield was reached with TRI (23.4%) than with ORY (8.3%), and in *E. rosea* a higher mortality was observed with TRI (41.6%) compared to ORY (19.6%).

In ORY treatments, a positive correlation between T-yield and concentration of ORY was found for all three genotypes, with an overall average from 5.5% up to 24.9% for 50 μM and 250 μM, respectively. In TRI treatments T-yield reached a plateau at 50 μM for *E. rosea* and *E. rubra* and at 150 μM for *E. illinita*. An increase in exposure time caused a decrease in T-yield (on average from 21.4 to 6.8% for 2–4 days, respectively) and an increase in mortality (on average from 6.8 to 30.2% for 2 to 4 days, respectively) in ORY treatments. Only for *E. illinita*, the effect on the mortality was reversed, with a small decrease in mortality from 14.4 to 8.9%. In TRI treatments, the exposure time did not cause changes in T-yield and mortality.

The ORY treatment that yielded the best results for all three *Escallonia* genotypes was 2 days of 250 μM ORY, resulting in a high T-yield (22.0–56.7%) and low corresponding mortality (0.0–4.9%). No best treatment for TRI could be identified, as all concentrations yielded the approximate same number of tetraploids. In the control treatment of *E. rosea* a limited number of tetraploids and mixoploids were found.

Some octaploids were generated for *E. illinita* in the treatments with 150 μM TRI for 2, 3, and 4 days, and with 150 μM ORY for 2 and 4 days. One octaploid *E. rosea* was recovered after 4 days in the presence of 50 μM TRI. The following treatments yielded one octaploid *E. rubra*: 4 days of 150 μM TRI, 2 days with 150 μM ORY, and 3 days with 50 μM TRI. Two octaploid *E. rubra* were recovered after 3 days with 250 μM ORY. The percentage mixoploids for the shock experiments with ORY reached up to 33.3%, and with TRI up to 31.7%. No mixoploids were retained for further evaluation, since sufficient tetraploid plants were available.

#### Continuous treatments

The resulting T-yield and mortality of the continuous treatments are shown in Figures 3C,D. Differences between TRI and ORY were observed. For all three species, TRI induced more tetraploids compared to ORY. This effect was most substantial in *E. illinita* (on average 5.5% T-yield with ORY, 32.3% with TRI). On average, ORY led to higher mortalities than TRI.

The T-yield reached a plateau at 5 μM ORY for *E. rosea*, and at 1 μM ORY for *E. rubra*. For *E. illinita*, the T-yield was positively correlated with the concentration. The mortality of all three species increased with ORY concentrations (on average from 11.6 to 38.7%). For TRI treatments, similar observations were made for both T-yield and mortality. For *E. rosea* and *E. rubra* the plateau in T-yield was reached at 5 μM TRI, while the T-yield of *E. illinita* increased with the concentration. No clear effect of the exposure time on the T-yield and mortality was found for both ORY and TRI. *E. rosea* and *E. rubra* showed a small decline in T-yield with increasing exposure and a small increase in mortality.

In this continuous treatment experiment, a 10 week exposure of 5 μM of TRI was the best treatment overall, despite species-dependent sensitivity toward the antimitotic agents used. For *E. rosea* and *E. rubra*, this resulted in 43.5 and 54.2% tetraploids, respectively. For *E. illinita*, 10 weeks on 5 μM of TRI resulted in a 63.4% T-yield, which was a close second to 8 weeks of 10 μM with a T-yield of 65.5%. The mortality of 10 weeks of 5 μM TRI of all three species was 12.5% or lower. For ORY, the best yielding treatment, or a close second best for *E. rosea*, was 8 weeks with 1 μM of ORY. However, the T-yield of the best ORY treatment was much lower or similar to the T-yield in the best TRI treatment.

Some continuous treatments yielded octaploids. One *E. illinita* octaploid was recovered after 10 weeks with 10 μM TRI. For *E. rosea*, all 10 and 6 week TRI treatments yielded a sum of 10 octaploids. For *E. rubra*, the following treatments yielded 14 octaploids together: 6 weeks of 1 μM and 5 μM ORY, 6 weeks of 1 μM and 10 μM TRI, 8 weeks of 5 μM and 10 μM TRI and 10 weeks of 5 μM TRI. The percentage mixoploids for the continuous experiments with ORY reached up to 31.0%, and with TRI up to 82.9%. No mixoploids were retained for further evaluation, since sufficient tetraploid plants were available.

In total, for both the shock and continuous experiments, 1,431 nodes of *E. illinita* were treated with ORY or TRI, with 218 tetraploid numbers created. For *E. rosea*, 195 tetraploids were induced on a total of 1,210 nodes, and for *E. rubra*, 221 tetraploids were obtained on 932 nodes.

### Characterization of the tetraploids

#### Rooting capacity

The rooting capacity after 5 weeks did not differ between the diploid and tetraploid group for any of the three species (Table 2). In addition, the root dry weight did not significantly differ between diploids and tetraploids.

**Table 2:** Rooting capacity and dry weight of cuttings of diploid (D) and tetraploid (T) *E. illinita, E. rosea*, and *E. rubra*.

***Escallonia* genotype**	**Ploidy group**	**Number of cuttings**	**Repetitions**	**Rooting (%)**	**Root dry weight (mg)**
*E. illinita*	D	136	2	94.9^NS^	1.11 ± 0.96^NS^
	T	126	2	96.1	1.06 ± 0.74
*E. rosea*	D	194	4	78.9^NS^	0.56 ± 0.44^NS^
	T	252	4	78.2	0.49 ± 0.31
*E. rubra*	D	188	4	80.3^NS^	1.66 ± 2.71^NS^
	T	192	4	79.2	1.84 ± 2.29

*Statistical differences per genotype between diploids and tetraploids (MWUT*,

NS *, not significant*;

*S, significant at p = 0.05)*.

#### Morphological traits

##### Growth and branching

For all three species, much phenotypic variation for each trait was present within both the diploid control group and the tetraploid group. The individual numbers from the homoploid groups were not always clearly separated: many diploid and tetraploid numbers had an intermediary morphology (Supplementary Figures 1–3). But when analyzing the average results, different trends could be observed (Table 3).

**Table 3:** New apical shoot length (NSL) and its internode length (NSIL), axillary branch length (BL) and its internode length (BIL), and the axillary budburst (% of buds on the main stem that sprouted) of *E. illinita, E. rosea*, and *E. rubra* diploid controls (D) and tetraploid numbers (T), measured on at least 4 four plantlets per plant number (mean ± standard deviation).

***Escallonia* species**	**Ploidy group**	**Analyzed plant numbers**	**NSL (cm)**	**NSIL (cm)**	**BL (cm)**	**BIL (cm)**	**Axillary budburst (%)**
*E. illinita*	D	6	11.0 ± 4.5^NS^	1.0 ± 0.2^***^	23.6 ± 2.6^*^	1.4 ± 0.1^*^	15.6 ± 2.0^**^
	T	23	11.4 ± 2.4	1.1 ± 0.2	20.1 ± 2.9	1.5 ± 0.1	18.0 ± 2.3
*E. rosea*	D	4	3.8 ± 2.8^***^	0.3 ± 0.1^***^	6.8 ± 2.2^***^	0.4 ± 0.1^***^	93.1 ± 10.3^**^
	T	6	1.3 ± 1.2	0.2 ± 0.2	3.4 ± 2.0	0.3 ± 0.1	85.1 ± 15.2
*E. rubra*	D	16	4.9 ± 4.3^*^	0.4 ± 0.3^***^	8.2 ± 2.1^***^	0.6 ± 0.1^***^	81.5 ± 17.0^**^
	T	23	5.5 ± 3.9	0.5 ± 0.3	8.6 ± 2.0	0.7 ± 0.1	76.2 ± 16.7

*Statistical analysis within the genotype between diploids and tetraploids with MWUT for pairwise comparison, except for NSL and NSIL of E. illinita and E. rubra, a T-test for pairwise comparison was used*.

NS *, Not significant*;

* *p = 0.05*;

** *p = 0.01*;

*** *p = 0.001*.

For *E. illinita*, the average length of the new apical shoot (NSL) did not differ significantly between diploids and tetraploids, yet its mean internode length (NSIL) increased significantly. In a further developed state (7 weeks later), tetraploids had a significantly higher axillary budburst (+2.4%) on the main stem than diploids. The presence of more branches in tetraploids caused a significant decrease in average branch length. The internode length of the axillary branches (BIL) was also significantly increased with ±0.1 cm in tetraploids, as was the case for the NSIL. Thus, for *E. illinita*, the added variation created a tendency for shorter branches but longer internodes.

For *E. rosea*, on average the tetraploids reached about half the length of diploids (Table 3). Both the NSL and the BL of tetraploids were significantly shorter. In addition, the internode length was significantly shorter in tetraploids than in diploids for both the NSIL and the BIL. The diploid axillary budburst decreased significantly by ±8% due to polyploidization. Polyploidization resulted in a one-directional change toward smaller and slower growing plants for *E. rosea*.

Tetraploids of *E. rubra* were larger and faster growing than the diploid controls (Table 3). The NSL increased significantly with 0.6 cm. The increase was smaller for BL (only ±0.4 cm) but highly significant. NSIL and BIL increased significantly. The axillary budburst of the diploid controls was significantly higher (5%) than of the tetraploid numbers. Therefore, it could be concluded that polyploidization resulted in a one-sided broadening or the variation present in the diploid group toward faster growing and taller plants.

##### Leaf morphology

For both *E. illinita* and *E. rubra*, chromosome doubling caused wider leaves and a larger leaf surface (Table 4). The leaves from tetraploid *E. illinita* numbers were significantly wider, but not longer than the leaves of diploid controls. This resulted in a significant decrease in length/width ratio (l/w) and in a significant increase in leaf surface of ±0.71 cm^2^. Tetraploid *E. rubra* leaves were significantly wider (±0.51 cm), but not longer than the leaves of diploids. This resulted in a significant decrease in l/w and a significant increase in leaf surface of ±1.14 cm^2^. In contrast, *E. rosea* tetraploid leaves were significantly reduced in both leaf length and width, resulting in a significant decrease of ±0.27 cm^2^ in leaf surface. The decrease was proportional for length and width, so no significant changes in l/w ratio were observed.

**Table 4:** Leaf length, width, length/width ratio (l/w), and leaf surface of diploid (D) and tetraploid (T) *E. illinita, E. rosea*, and *E. rubra*, collected on at least 4 four plantlets per plant number (means ± standard deviation).

***Escallonia* genotype**	**Ploidy group**	**Analyzed plant numbers**	**Number of leaves**	**Length (cm)**	**Width (cm)**	**l/w ratio**	**Leaf surface (cm^2^)**
*E. illinita*	D	6	390	4.17 ± 0.70^NS^	1.75 ± 0.31^***^	2.40 ± 0.25^***^	4.49 ± 1.32^***^
	T	23	1,270	4.12 ± 0.83	2.10 ± 0.46	1.98 ± 0.24	5.20 ± 1.99
*E. rosea*	D	4	180	2.55 ± 0.73^**^	0.92 ± 0.32^**^	2.92 ± 0.64NS	1.63 ± 0.88^***^
	T	6	320	2.33 ± 0.66	0.85 ± 0.33	2.87 ± 0.60	1.36 ± 0.82
*E. rubra*	D	16	450	4.27 ± 0.83^NS^	1.70 ± 0.35^***^	2.53 ± 0.36^***^	4.80 ± 1.80^***^
	T	23	1,259	4.26 ± 0.81	2.21 ± 0.47	1.96 ± 0.25	5.94 ± 2.14

Statistical analysis within the genotype between diploids and tetraploids (

NS *, Not significant*;

** *p = 0.01*;

*** *p = 0.001*.

*Pairwise comparison with the Mann-Whitney U-Test for E. rosea and E. rubra, T-Test for E. illinita*.

##### Flower characteristics

**Figure 4:**
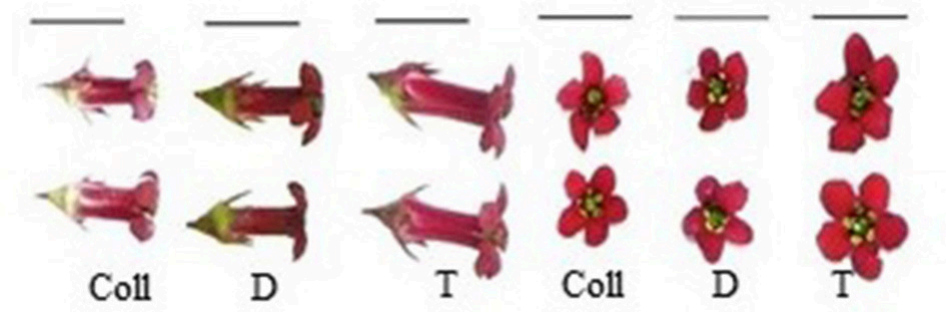
Side view and top view of flowers of *E. rubra* from the plant in our collection (Coll), from the diploid controls (D), and the tetraploid numbers (T) in the field. (bar = 1 cm).

At the time of this writing, tetraploid and diploid genotypes of *E. rosea* and *E. illinita* have not flowered. The diploid controls (D), the plants in our collection (Coll) and the tetraploid numbers (T) of *E. rubra* started flowering by mid-June (Figure 4). Flowers from the diploid control plants were significantly shorter (D: 0.96 ± 0.06 cm; Coll: 0.99 ± 0.06 cm) and wider (D: 0.28 ± 0.03 cm; Coll: 0.27 ± 0.03 cm) than flowers from the genotype of *E. rubra* in the collection (*p* = 0.001 and 0.01 respectively). The corolla surface (D: 0.31 ± 0.06 cm^2^; Coll: 0.30 ± 0.04 cm^2^) was not significantly different between diploid controls and the collection genotype. As shown in Figure 4, the flowers of the tetraploid group were significantly larger and wider than both the collection and diploid flowers (T: 1.18 ± 0.11 cm long and 0.35 ± 0.03 cm wide) (*p* = 0.001). The corolla surface of the tetraploid flowers (0.47 ± 0.08 cm^2^) was significantly larger than the corolla surface of both the collection and diploid flowers (*p* = 0.001).

#### Plant architecture

The results from the image analysis of *E. rosea* and *E. rubra* is shown in Table 5. The tetraploids of *E. rosea* were much smaller than the diploids. The average plant area decreased significantly by ±60% in top view and with ±33% in side view. The plant width and height of tetraploids decreased significantly. However, the percentage of the area the plant occupies in the bounding rectangle (BR) was significantly larger in tetraploids than in diploids. This was also shown by the percentage of the minimal bounding circle (MBC) filled by the plants, where tetraploids filled a significantly larger area. In addition, the circularity of the tetraploids was significantly larger than the diploids. These data showed that the tetraploid numbers were not only smaller in area, height and width, but they were much denser and less spindly than the diploids. The variation within individual diploids and tetraploids is shown in Supplementary Figure 4.

**Table 5:** Plant area, circularity of the convex hull and the % filled by the plant in the minimal bounding circle (MBC) from the overhead images, and the plant area, width, height, and the % of the bounding rectangle (BR) filled by the plant from the side view images, measured on at least 4 plantlets per number (mean ± standard deviation).

***Escallonia* species**	**Ploidy group**	**Analyzed plant numbers**	**Top view**			**Side view**			
			**Plant area (cm^2^)**	**Circularity^a^**	**% of MBC filled**	**Plant area (cm^2^)**	**Plant width (cm)**	**Plant height (cm)**	**% of BR filled**
*E. rosea*	D	4	319.1 ± 141.4^***^	0.76 ± 0.08^**^	12.6 ± 4.7^***^	136.5 ± 59.0^***^	47.7 ± 15.2^***^	16.6 ± 8.5^**^	18.6 ± 4.4^***^
	T	6	129.7 ± 102.1	0.81 ± 0.07	24.5 ± 8.7	91.7 ± 48.7	29.0 ± 12.1	12.5 ± 5.2	26.3 ± 6.2
*E. rubra*	D	16	394.1 ± 110.7^NS^	0.75 ± 0.09^***^	12.1 ± 3.3^***^	191.0 ± 54.6^*^	58.2 ± 14.5^***^	21.0 ± 8.8^NS^	17.9 ± 6.0^*^
	T	23	371.5 ± 86.8	0.46 ± 0.08	15.0 ± 4.8	180.4 ± 49.2	50.1 ± 13.3	20.8 ± 7.8	19.5 ± 6.5

*Statistical analysis within the genotype between diploids and tetraploids with MWUT for pairwise comparison*.

NS *, Not significant*;

* *p = 0.05*;

** *p = 0.01*;

*** *p = 0.001*.

a Circularity = 4 ^*^ π ^*^ area of convex hull/(perimeter of convex hull)^2^.

For *E. rubra*, the average plant area viewed from the side decreased significantly (±6%) in tetraploids compared to diploids (Table 5), due to a reduction in plant width of ±14%. The plant height and plant area in top view did not change. The circularity of tetraploids was significantly lower than in diploids. The percentage in which the area of the MBC and the BR are filled by the plants both increase significantly.

#### PCA of the phenotyping traits

**Figure 5:**
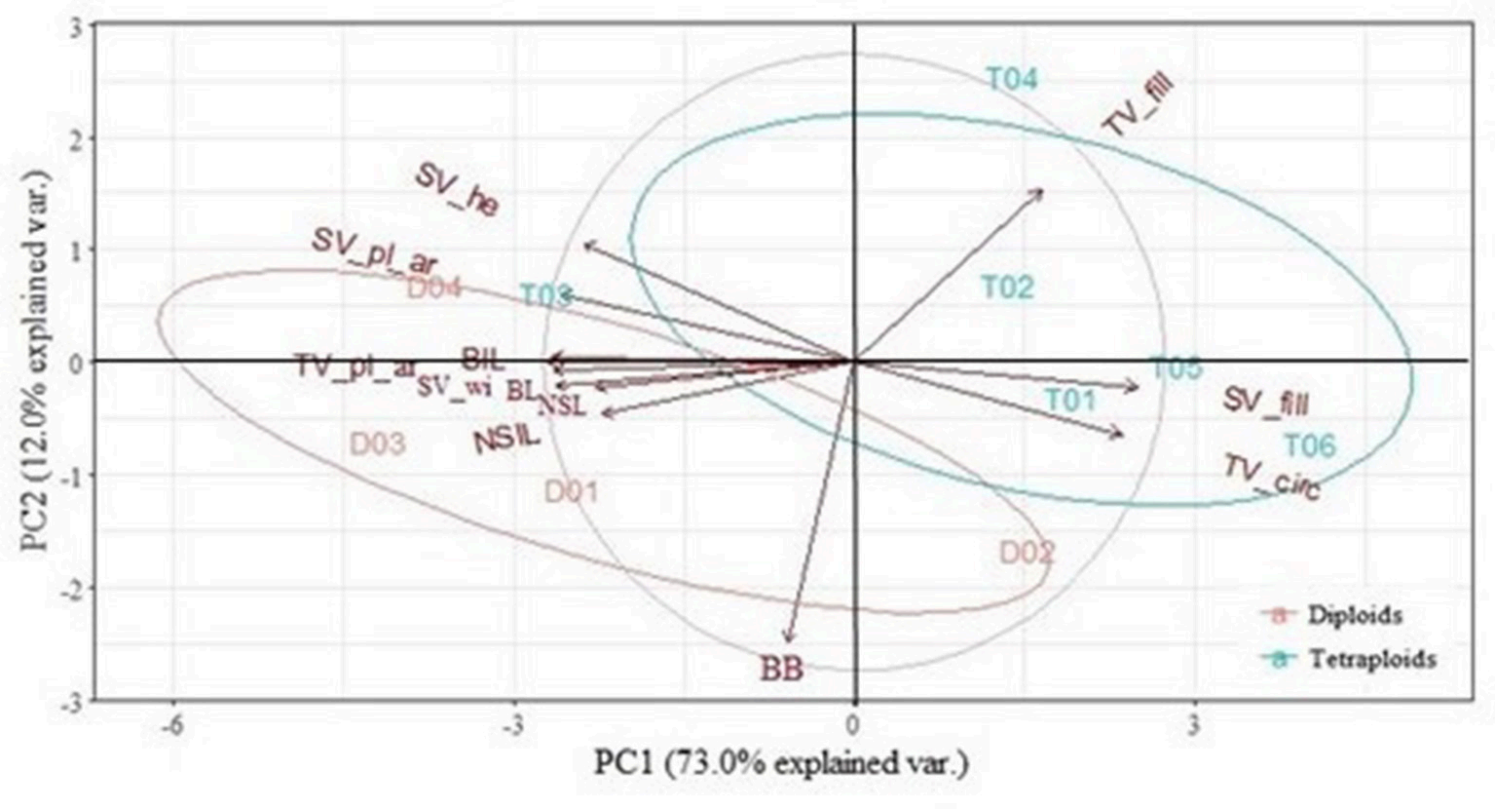
Principal Component Analysis (PCA) of the diploids (D) and tetraploids (T) of *Escallonia rosea*. 2 PCs with an eigenvalue higher than one, explained 85.0% of the variation. NSL, new shoot length; NSIL, new shoot internode length; BB, axillary budburst; BL, branch length; BIL, branch internode length; TV_pl_ar, plant area in top view; TV_circ, circularity; TV_fill, % filled of the minimal bounding circle in top view; SV_pl_ar, plant area in side view; SV_he, plant height in side view, SV_wi, plant width in side view; SV_fill, % filled of the bounding rectangle in side view.

PCA of *E. rosea* on 12 traits could be reduced to 2 PCs, explaining 85.0% of the variation (Figure 5). PC1 was mostly determined by the positively correlated SV_fill and TV_circ (0.897 and 0.856) and by the negatively correlated NSL, NSIL, BL, BIL, TV_pl_ar, SV_pl_ar, SV_he, and SV_wi (−0.822, −0.783, −0.979, −0.968, −0.980, −0.945, −0.885, and −0.962 respectively). The diploid and tetraploid group were mainly separated by BB and TV_fill. However, if we do not take the deviating phenotypes of T03 and D02 into account, than any of the used traits could be used as group separators.

**Figure 6:**
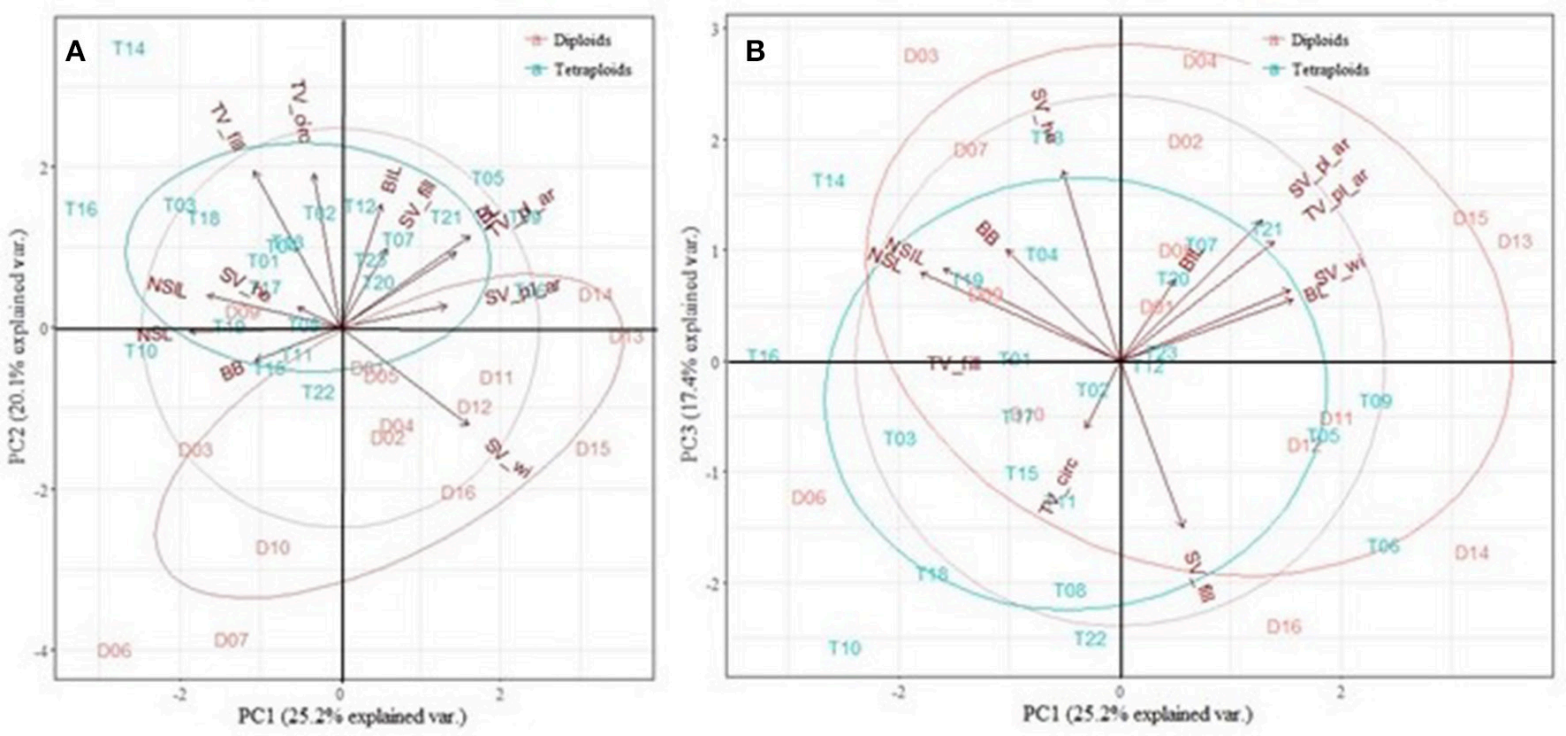
Principal Component Analysis (PCA) of the diploids (D) and tetraploids (T) of *Escallonia rubra*. 5 PCs with an eigenvalue larger than one, explained 84.5% of the variation. **(A)** PC1 vs. PC2, with 45.3% of the variance. **(B)** PC1 vs. PC3, with 42.6% of the variance. NSL, new shoot length; NSIL, new shoot internode length; BB, axillary budburst; BL, branch length; BIL, branch internode length; TV_pl_ar, plant area in top view; TV_circ, circularity; TV_fill, % filled of the minimal bounding circle in top view; SV_pl_ar, plant area in side view; SV_he, plant heigth in side view; SV_wi, plant width in side view; SV_fill, % filled of the bounding rectangle in side view.

For *E. rubra*, five PCs had an eigenvalue higher than 1, which explains 84.5% of the variance between all the homoploid groups. PC1 was positively correlated with SV_wi (0.730) and negatively correlated with NSL, NSIL, and TV_fill (−0.673, −0.678, and −0.617 respectively). PC2 was mostly determined by the positively correlated traits BL, BIL, TV_circ and TV_fill (0.636, 0.692, 0.667, and 0.628 respectively). In Figure 6A, a clear separation between diploids and tetraploids was made by TV_fill, TV_circ and SV_wi. PC3 was determined positively by SV_he (0.686) and negatively by SV_fill (−0.653). Comparing PC1 to PC3 (Figure 6B), group separation was mainly caused by SV_pl_ar and TV_pl_ar, and to a minor extent by SV_wi and BL. The most distinguishing trait to determine PC4 was the positively correlated SV_he (0.621) and for PC5 BIL (−0.525) and SV_pl_ar (0.563). Although PC4 and PC5 explained 11.9 and 10.5% of the variance, respectively, no separation was made between the diploid and the tetraploid group with these components.

**Figure 7:**
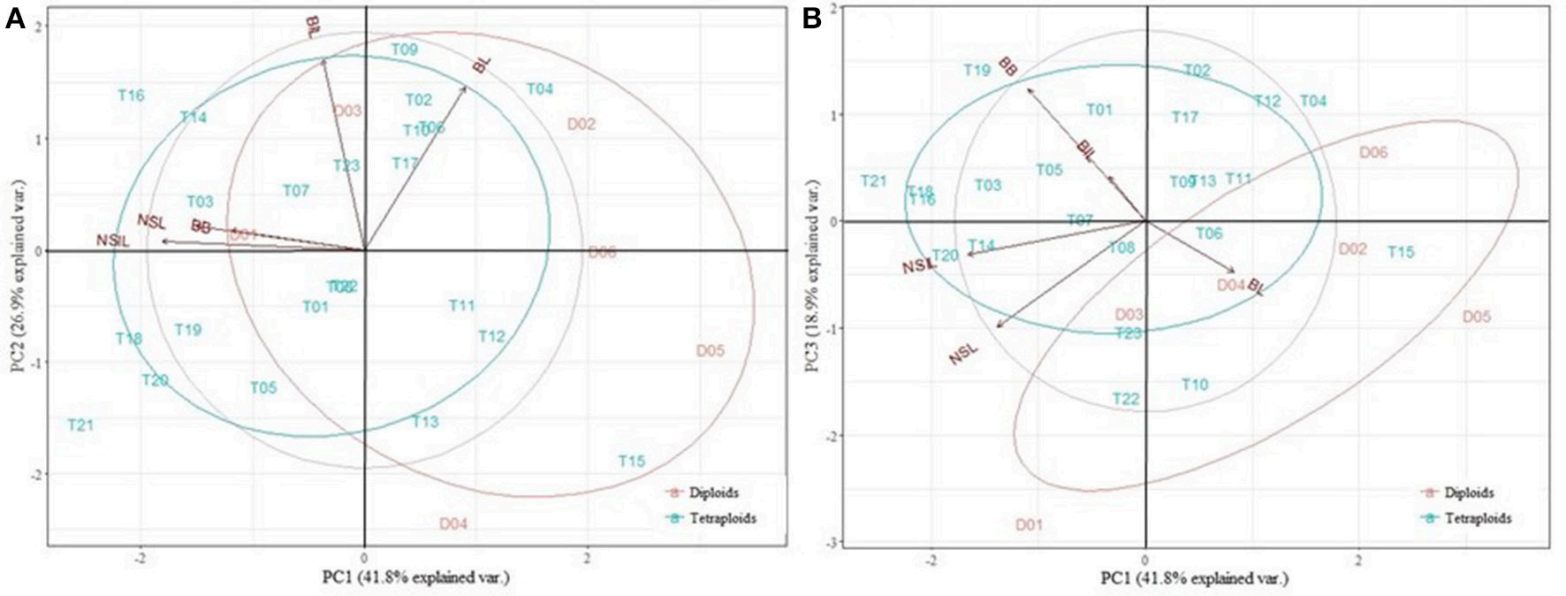
Principal Component Analysis (PCA) of the diploids (D) and tetraploids (T) of *Escallonia illinita*. 3 PCs with an eigenvalue larger than 1 explained 87.6% of the variance presence. **(A)** PC1 vs. PC2, explaining 68.7% of the variance. **(B)** PC1 vs. PC3, explaining 30.7% of the variance. NSL, new shoot length; NSIL, new shoot internode length; BB, budburst; BL, branch length; BIL, branch internode length.

The PCA analysis on *E. illinita* was conducted for five morphological traits, namely NSL, NSIL, BL, BIL, and BB. Three principal components had an eigenvalue larger than one, explaining 87.6% of the variance present (Figure 7). PC1 was mostly determined by the negatively correlated NSL, NSIL, and BB (−0.730, −0.925, and −0.611 respectively); traits explaining PC 2 were BL and BIL (0.747 and 0.877, respectively). PC3 was determined mostly by one trait, namely BB (0.681). As shown in Figure 7A, separation between the diploid and tetraploid group by comparing PC1 and PC2 was mainly caused by NSL and NSIL and to a lesser amount by BB. When comparing PC1 and PC3 (Figure 7B), group separation was caused by BB, and only marginally by BIL and BL.

#### Cold tolerance

**Figure 8:**
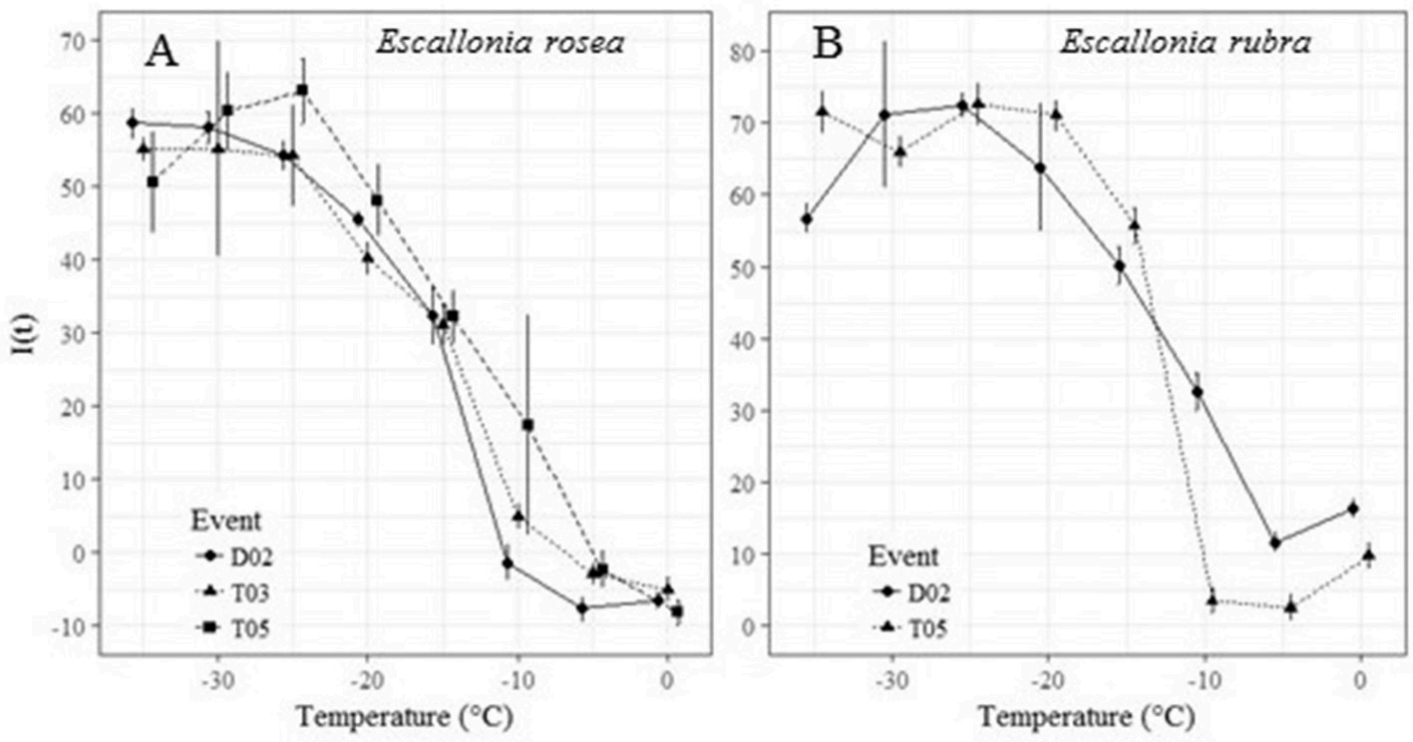
The Index of injury [I(t)] of the controlled freezing test for **(A)** 1 diploid (D02) and 2 tetraploid (T03 and T05) numbers of *E. rosea*. and **(B)** 1 diploid (D02) and 1 tetraploid (T05) number of *E. rubra*, with the calculated LT50 values.

The index of injury for *E. rosea* and *E. rubra* for the analyzed temperatures are shown in Figure 8. For *E. rosea*, 1 diploid (D02) and 2 tetraploids (T03 and T05) were analyzed in the controlled freezing test (Figure 8A). Polyploidization had no effect on the calculated LT50: −15.1 and −13.8°C for T03 and T05, respectively, compared to −14.6°C for the diploid D02.

In contrast to *E. rosea*, a positive effect of polyploidization on cold tolerance was observed in *E. rubra*. The LT50 for T05 of *E. rubra* was significantly lower compared to D02, namely −11.8°C vs. −7.7°C, respectively (Figure 8B).

## Discussion

### Morphological and genetic variation within the *Escallonia* collection

Correct identification of available germplasm and knowledge on phylogenetic relatedness between different accessions are important for plant breeding. The accessions were verified using morphological descriptions, AFLP analysis and (cyto)genetic information. The phylogenetic tree (Figure 2) showed that *E. pendula* was the most distantly related species. This concurs with the results of Sede et al. (2013), who created a phylogenetic tree of *Escallonia* spp. using plastid DNA. *E. pendula* was also the only tetraploid species. Several genotypes with the same name from different accession sites displayed differences in morphology. Based on morphological descriptions *E. alpina* (2) from the Tree Nursury of Rein and Mark Bulk (BRMB, Boskoop, The Netherlands) was a true *E. alpina* (Krüsmann, 1960; Bean and Murray, 1989; Hilliers Garden, 1991), while *E. alpina* (1) from Hillier Garden (HG, Ampfield, United Kingdom) had a deviating flower color (white instead of red). *E. illinita* (2) from the Botanical Garden from the University of Vienna (BGUW, Vienna, Austria) was a true *E. illinita*, because the genotype concurred best with the description in literature (Krüsmann, 1960; Bean and Murray, 1989; Hilliers Garden, 1991). *E. illinita* (1) from Tree Nursery De Neve (DN, Oosterzele, Belgium) resembles the phenotype of *E. illinita* (2) very closely, except for the flower color, which is pink instead of white, which might be the result of a natural hybridization or a mutation. The genotypes *E*. “Iveyi” and *E. iveyi* (x) [ = (*E. rosea* × *E. rubra*) × *E. bifida*] (Krüsmann, 1960; Bean and Murray, 1989; Hilliers Garden, 1991) were most likely the same genotype from a different acquisition site from Tree Nursery De Neve (DN, Oosterzele, Belgium) and the Botanical Garden Meise (Meise, Belgium) respectively, since they clustered very closely together (Figure 2).

Many natural *Escallonia* hybrid cultivars are described, indicating an easy interspecific hybridization. Data of chromosome counts showed that all but one species have 24 chromosomes. Diploid genome sizes varied between 1.06 ± 0.02 pg/2C for *E*. “Langleyensis” and 1.43 ± 0.03 pg/2C for *E. bifida*. No information was found about crosses between these latter two species. However, *E*. “Iveyi” [or *E. iveyi* (x)] is a hybrid of [ = (*E. rosea* × *E. rubra*) × *E. bifida*], and the genome sizes of *E. rosea* and *E. rubra* were also rather small (1.11 ± 0.06pg/2C and 1.07 ± 0.06 pg/2C respectively), while the genome size of *E. bifida* is about 30% bigger. The hybrid *E*. “Iveyi” and *E. iveyi* (x) had 2C values of 1.32 ± 0.16 and 1.25 ± 0.03 pg/2C, respectively, in between the parental genome sizes. This indicates that the variation in genome size is not always a barrier for interspecific hybridization. The Kew C-Values database contains information on *E. rubra*, 0.85 pg/2C measured with Feulgen densitometry and on *E. pulverulanta*, 1.13 pg/2C measured with flow cytometry with PI (Kew Botanical Garden, 2001). *E. rubra* in our collection had a 2C value of 1.07 ± 0.06 pg, measured with flow cytometry with PI. The difference in analyzing method could account for the difference in 2C value (Moscone et al., 2003). The 2C value of *E. pulverulenta* with PI flow cytometry as published by Kew Botanical Garden (2001) is within the range of the values that resulted from our analysis. No data of crosses between the tetraploid *E. pendula* and any of the diploid genotypes was found, so no information on interploidy crosses was available.

### *In vitro* continuous treatment with trifluralin is most efficient to induce tetraploids in *Escallonia*

The best treatments to apply on other *Escallonia* genotypes were 2 days of 250 μM ORY and 10 weeks of 5 μM TRI. These treatments yielded most tetraploids: on average across all three species, they produced 39.2 and 53.7% tetraploids, respectively. Moreover, these treatments also displayed low mortalities of 3.3 and 5.6%, respectively. Compared to similar studies (Dhooghe et al., 2011), the chosen polyploidization treatments were highly efficient with low mortalities. In mandarins, a submersion of shoot tips in a colchicine solution yielded no tetraploids, and only in some cases a few chimeras (2x-4x), while the mortalities ranged between 20 and 100% (Aleza et al., 2009). A shock treatment with oryzalin on nodal segments of *Rosa rugosa* showed mortalities between 25 and 80%, but a tetraploid yield of 44% was reached (Allum et al., 2007). Nodal segments of *Rosa* “Thérèse Bugnet” were exposed to 5 μM of oryzalin on solid medium for 1–3 days. A tetraploid yield of 67% was reached, but with high mortalities of 80–100% (Kermani et al., 2003).

In all shock treatments, the mortality was erratic and quite high, even in control treatments, indicating a high stress level caused by the shaking treatment and the addition of EtOH or acetone as solvents for ORY and TRI. As a consequence of that stress, several tetraploids and mixoploids were recovered in the control treatment of *E. rosea*. High stress levels can cause endopolyploidy or endoreduplication (Barrow and Jovtchev, 2007). Spontaneous induction of polyploid plants during *in vitro* regeneration has also been reported in *Phalaenopsis* (Chen et al., 2009) and in *Gentiana* (Tomiczak et al., 2015). Given that the stress induced by the shock treatment caused great variation in mortality, we could conclude that the continuous treatment was most stable.

Octaploid *Escallonia* were recovered in all three species and for both mitotic agents from several treatments. Trifluralin induced more octaploids compared to oryzalin: in total, 1.7 and 0.5%, respectively, or 29 and 9 octaploids for all three species across all experiments. Long exposure times can lead to a redoubling to produce octaploids (Allum et al., 2007; Dhooghe et al., 2011). However, no octaploids could be acclimatized due to poor viability and lack of growth vigor. This unfavorable effect of higher ploidy levels has also been observed in octaploid *Rosa rugosa* hybrids (Allum et al., 2007).

Differences in T-yield and mortality were observed between ORY and TRI, while these mitotic inhibitors have the same mode of action (Dhooghe et al., 2011). A preliminary study with *E. rosea* and *E. rubra* showed a very low T-yield with colchicine compared to ORY and TRI, even though 10x higher concentrations of colchicine were used (Denaeghel et al., 2015). Colchicine, ORY, and TRI are all metaphase inhibitors. They disturb the formation of the spindle, which is essential for polar migration of the homologous chromosomes to the daughter cells (Dewitte and Murray, 2003). The difference in T-yield when using different mitotic inhibitors might be due to differences between the antimitotica in solubility, in penetration or transportation in the plant tissue, but also in sensitivity among the *Escallonia* genotypes.

### Changes in plant morphology and physiology after polyploidization

Three *Escallonia* species were polyploidized, and species dependent effects on the plant growth and architecture were observed. Our results indicate that no phenotypic predictions on the outcome of a polyploidization experiment can be made, as every species has to be evaluated separately. This interaction between genetic background and ploidy level was also demonstrated by Riddle et al. (2006) who studied the effect of polyploidization on the phenotype of 1x, 2x, and 4x plants of four *Zea mays* cultivars.

Plant architecture determines the visual attractiveness, an important criterion for the commercial success of ornamental plants (Li-Marchetti et al., 2015). The architecture of a plant consists of the relative arrangement of each of its parts. Four major categories can be distinguished: (1) branching process, (2) growth process, (3) the morphological differentiation of axes, and (4) the position of reproductive structures (Barthelemy and Caraglio, 2007). In the present study the branching process and growth process were analyzed for tetraploid and diploid *Escallonia* genotypes.

An increased budburst or axillary branching on the main stem was obtained in tetraploid *E. illinita*, while both *E. rubra* and *E. rosea* displayed a decrease in axillary budburst in tetraploids. The branching is controlled by the apical dominance, while the shoot apex controls the outgrowth of axillary buds (Cline, 1994). Several hypotheses attempt to explain branching control by apical dominance, but a common factor appears to be the levels of auxin and cytokinin, or the sensitivity of the plant tissues toward these hormones (Dun et al., 2006). If polyploidization influences the production, transport or sensitivity toward auxin or cytokinin, changes in apical dominance can occur. A lower apical dominance leads to a higher axillary budburst and more branches, i.e., a higher number of nutrient sinks. The same amount of nutrients that normally would go to the outgrowth of the apical shoot is divided among the branches, leading to a slower growth of each of the branches compared to the apical shoot (Dun et al., 2006). This process is applied artificially by growers of ornamentals by frequent mechanical or chemical pruning, or by applying plant hormones exogenously (Meijon et al., 2009; Mutlu and Kurtulan, 2015). To analyze the growth process, the primary growth of the rooted cutting and the outgrowth of axillary branches after pinching were measured and the internodal distance was determined. Clearly, both results of branching and internode length were necessary to interpret the effect on the overall plant size. The increase in axillary budburst for tetraploid *E. illinita* could potentially cause more, shorter branches to grow. However, an increased internode length reduced the effect of the increased axillary budburst on the compactness, resulting in only slightly shorter branches. The combination of a decrease in both axillary budburst and internode length led to a large decrease in size and plant area in tetraploid *E. rosea*. A decrease in plant height by reducing internode length after polyploidization was also observed in garden petunia (Regalado et al., 2017), in *Rosa multiflora* (Feng et al., 2017), in *Platanus* (Liu et al., 2007), in *Thymus* (Tavan et al., 2015), and in *Eriobotrya* (Blasco et al., 2015). In tetraploid *E. rubra*, chromosome doubling caused both a significant increase in internode length and a decrease in axillary budburst. The overall effect was an increased plant size and a looser plant habitus, as shown by the decrease in circularity. A similar increase in plant size was found in *Vitex* (Ari et al., 2015) and in some *Rosa* genotypes (Kermani et al., 2003).

Image analysis of *E. rosea* and *E. rubra* confirmed the effects on plant habitus as determined by measuring on the plants themselves, and added some information regarding visual attractiveness, such as circularity and the bushiness. In *E. rosea*, where a significantly lower axillary budburst in tetraploids could indicate less dense plants, the circularity and the % filled by the plant of the MBC and the BR clearly showed more circular and dense plants. For *E. rubra*, the image analysis showed no increase in size, except for plant width. The increase in the % of area of the MBC or BR that was filled by the plant, was likely caused by an increase in leaf area. The large decrease in circularity was caused by a polar outgrowth of branches on the main axis in tetraploids. This is not a desired characteristic by growers or consumers. It is clear that the image analysis adds valuable information about the plant architecture that could not be derived from the measurements of plant height, internode length and axillary budburst. Traits such as plant area and circularity would have been very difficult to determine correctly by means of visual scoring. This type of 2D image analysis has several advantages. It is non-invasive and could be repeated to analyze the growth dynamic over time (Fiorani and Schurr, 2013). Free, open-source software, such as ImageJ, is available to create a customized program for analysis. The correct analysis of the plant area, however, can be biased by overlapping or curling leaves when the image is taken from only one view (e.g., overhead view) (Humplik et al., 2015b). This was a problem with tetraploid *E. rubra*, where a decrease in plant width and axillary budburst should have resulted in a decreased plant area in top view, but was counteracted by an increased leaf area. Therefore, side view images from different angles were included. The efficiency of this image analysis could be optimized by determining the number of pictures in side view necessary to obtain the required information. A high-throughput phenotyping study on cereals and pea only included two side view images, rotated 90° vertically (Golzarian et al., 2011; Humplik et al., 2015a), which could reduce the time needed to take the images.

A PCA was conducted on the traits resulting from the growth and branching analysis and the image analysis in order to find the traits that are most important in discerning diploids from tetraploids. For *E. rosea*, the homoploid groups could be separated by BB and TV_fill. However, two highly deviant phenotypes were present, namely D05 and T03. If these numbers were not present in the PCA, all traits analyzed could be used to discern the homoploid groups. This indicates that for *E. rosea*, polyploidization causes changes in all morphological and architectural traits analyzed. For *E. rubra*, the most discerning traits were TV_fill, TV_circ, SV_wi, SV_pl_ar and TV_pl_ar. Unfortunately, no image analysis was done for *E. illinita*, and the PCA was made with only the growth and branching parameters measured on the plantlets. In this case, NSL, NSIL and BB were discerning traits. Globally, for each of the species, either BB or TV_fill is important, and only analyzing these two traits could help in separating the homoploid groups if other ploidy detection methods, e.g., flow cytometry, are not available. However, these traits alone are not capable of determining the compactness of the plants.

In all analyzed genotypes, the tetraploids broadened the phenotypic variation that was already present in the original diploid phenotype (Supplementary Figures 1–5). A genome-dosage effect is considered as a major contributor to the added variation by polyploidization. However, this usually leads to more intermediate phenotypes, and only for some alleles to extreme phenotypes (Osborn et al., 2003). This effect has mostly been studied on an evolutionary scale for natural auto- or allo-polyploids, and only occasionally in the first generation of synthetic autopolyploids.

Beside plant architecture, foliage and flower morphology are important characteristics for visual attractiveness of an ornamental. An increase in organ size is quite common after polyploidization (Dhooghe et al., 2011). The observed leaf morphology changes after polyploidization differed for the three *Escallonia* genotypes. Tetraploid *E. illinita* and *E. rubra* showed wider leaves than diploids, thus a decrease in l/w ratio and increase in leaf surface. An increase in leaf size due to *in vitro* chromosome doubling has been observed in *Rosa* (Allum et al., 2007; Feng et al., 2017) and in *Vitex* (Ari et al., 2015), and after *in vivo* chromosome doubling in *Ziziphus* (Shi et al., 2015). A decrease in l/w ratio was also observed in *Spathiphyllum* (Van Laere et al., 2010). A decrease in leaf area, as was the case for *E. rosea*, was similarly present in tetraploid apple (Hias et al., 2017) and in *Buddleja globosa* (Van Laere et al., 2011b). Flowers of *E. rubra* tetraploids were larger than flowers of the diploid counterparts. An increase in flower size was reported for *Rosa* (Allum et al., 2007), *Vitex* (Ari et al., 2015), and *Paulownia* (Tang et al., 2010).

Rooting capacity, which is important for commercial propagation, was not affected by polyploidization. A delay in rooting and a decreased root length has been reported previously in tetraploid *Thymus* (Tavan et al., 2015), but for *Escallonia*, this was not the case. Changes in stress resistance are often a consequence of chromosome doubling (Levin, 2002; Van Laere et al., 2010; Regalado et al., 2017). Since winter hardiness is an issue for *Escallonia* breeders, a cold tolerance test was conducted. Polyploidization did not have a negative effect on the cold tolerance of the tetraploids, and even an increase in cold tolerance was measured for tetraploid *E. rubra*. According to Hoffman and Ravesloot (1998) *E. rubra* can survive up to −12.2°C. However, this manual on nursery plants does not indicate how this value is achieved, and no further literature has been found. For *E. rosea*, no data on winter hardiness could be found, therefore our data could not be compared to literature.

## General conclusions

In this study, an efficient polyploidization protocol for the studied *Escallonia* species was set up, and tetraploids were characterized for their morphological traits and plant architecture. For both *E. illinita* and *E. rosea*, more compact phenotypes were obtained, but further field evaluations are needed to evaluate larger plants. In addition, both rooting and cold tolerance for the tetraploids scored at least as good as the original diploid genotypes. Therefore, we conclude that polyploidization is an efficient breeding tool to induce useful variation in *Escallonia*. The results of the image analysis added valuable information on the compactness and visual attractiveness of the plants, which would be hard to quantify with the standard morphological measurements such as shoot length and number of branches.

## Author contributions

Study conception and design: HD, KV, LL, PL, JV, and M-CV. Acquisition of data: HD. Analysis and interpretation of data: HD, KV, LL, PL, and M-CV. Drafting of manuscript: HD and KV. Critical revision: KV, LL, JV, and M-CV.

### Conflict of interest statement

The authors declare that the research was conducted in the absence of any commercial or financial relationships that could be construed as a potential conflict of interest.

## Abbreviations

BAP: 6-benzylaminopurine
BL: Branch Length
BIL: Branch Internode Length
BR: bounding rectangle
CoHu: convex hull
Coll: collection
D: Diploid
DAPI: 4′6-diaminidino-2-phenylindole
EC: electrical conductivity
EtOH: ethanol
I(t): Index of Injury at a given temperature t
MBC: minimal bounding circle
MS: Murashige & Skoog medium
NAA: 1-naphtaleneacetic acid
NSL: new shoot length
NSIL: new shoot internode length
ORY: oryzalin
PCA: principal component analysis
PC: principal component
PI: propidium iodide
T: tetraploid
TRI: trifluralin
TV: top view
TV_circ: plant circularity in top view
TV_fill: % of the area of the minimal bounding circle that was filled by the plant
TV_pl_ar: plant area in top view
T-yield: tetraploid yield
SV: side view
SV_fill: % of the area of the bounding rectangle was filled by the plant
SV_he: plant height in side view
SV_pl_ar: plant area in side view
SV_wi: plant width in side view.
