# Supplementary figures and images for: The Variable Effect of Polyploidization on the Phenotype in Escallonia
Source: Front Plant Sci. 2018 Mar 20;9:354. doi: 10.3389/fpls.2018.00354 (PMC5869194; doi:10.3389/fpls.2018.00354)

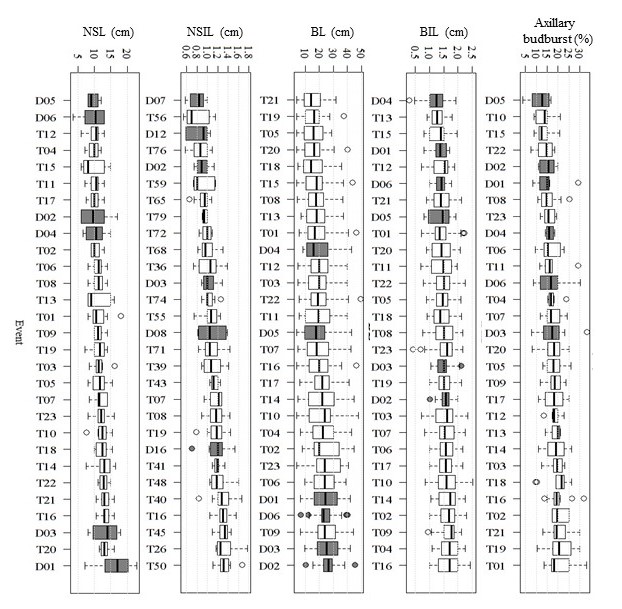

Supplement: Supplementary file 1 [file Image1.JPEG]

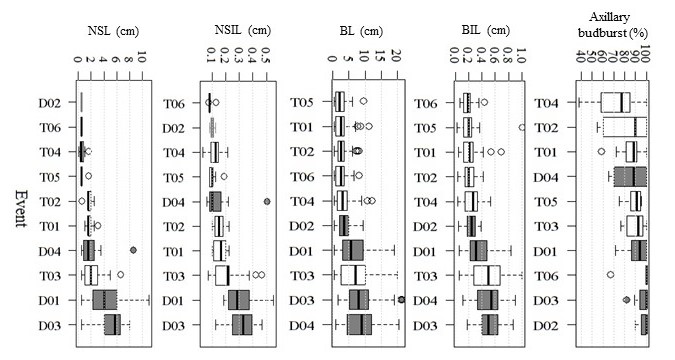

Supplement: Supplementary file 2 [file Image2.JPEG]

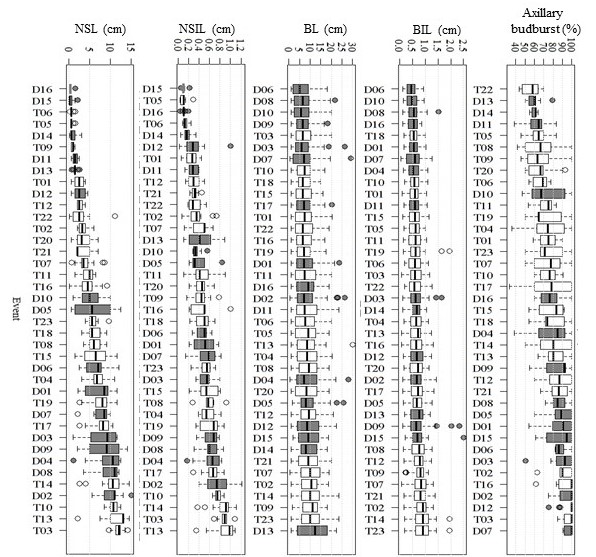

Supplement: Supplementary file 3 [file Image3.JPEG]

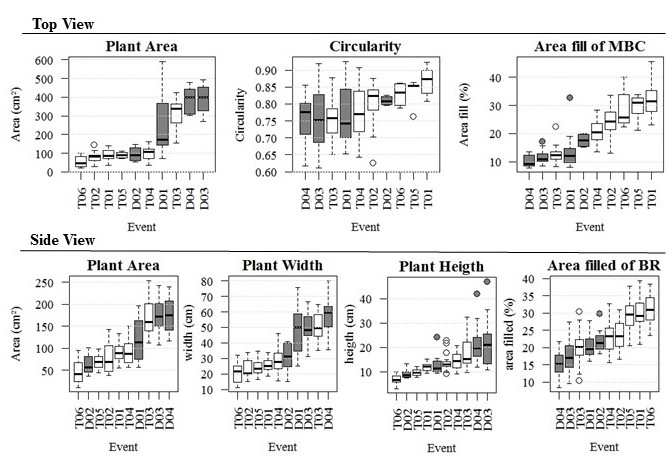

Supplement: Supplementary file 4 [file Image4.JPEG]

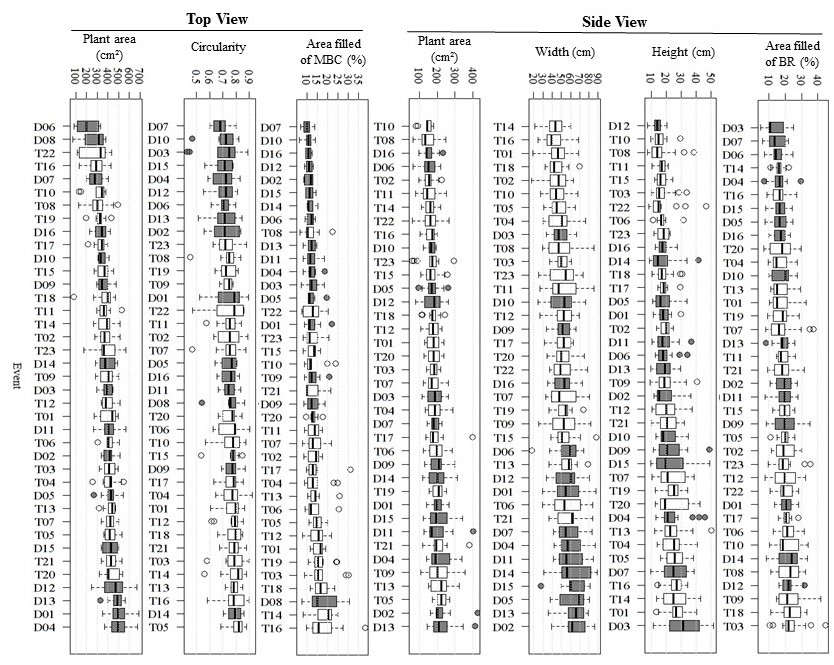

Supplement: Supplementary file 5 [file Image5.JPEG]
