# Supplementary material for: The Variable Effect of Polyploidization on the Phenotype in Escallonia
Source: Front Plant Sci. 2018 Mar 20;9:354. doi: 10.3389/fpls.2018.00354 (PMC5869194; doi:10.3389/fpls.2018.00354)
Supplement: Supplementary file 6 [file DataSheet1.docx]

Supplementary Material

The variable effect of polyploidization on the phenotype in *Escallonia*

Hanne Denaeghel^1,2*^, Katrijn Van Laere^1*^, Leen Leus^1*^, Peter Lootens^1*^, Johan Van Huylenbroeck^1*^, Marie-Christine Van Labeke^2^

^1^ Applied Genetics and Breeding, Plant Sciences Unit, Flanders Research Institute for Agriculture, Fisheries and Food (ILVO), Melle, Belgium

^2^ Department of Plant Production, Faculty of Bioscience Engineering, Ghent University, Ghent, Belgium.

*** Correspondence:**Hanne Denaeghel
[hanne.denaeghel@ilvo.vlaanderen.be](mailto:hanne.denaeghel@ilvo.vlaanderen.be)

Katrijn Van Laere

[Katrijn.vanlaere@ilvo.vlaanderen.be](mailto:Katrijn.vanlaere@ilvo.vlaanderen.be)

**
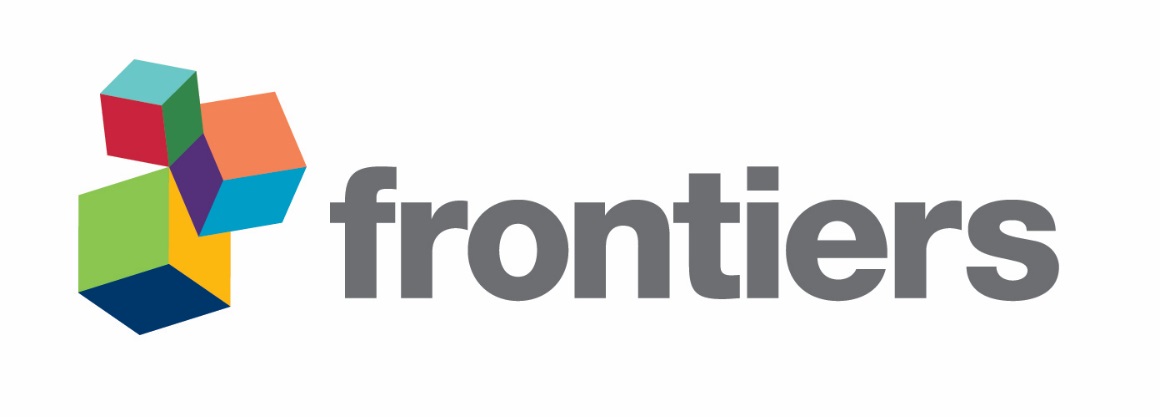
**

# Supplementary Figures

**Supplementary Figure 1:** The length of the new apical shoot (NSL) and its internode length (NSIL) of the 5 week old rooted cuttings. The length of the branches (BL), their internode lengths (BIL), and the axillary budburst (%) of the pinched plantlet 17 weeks after cutting of diploid (D) and tetraploid (T) numbers of *Escallonia illinita* (Diploid = grey, Tetraploid = white).

**Supplementary Figure 2**: The length of the new apical shoot (NSL) and its internode length (NSIL) of the 5 week old rooted cuttings. The length of the branches (BL), their internode lengths (BIL) , and the axillary budburst (%) of the pinched plantlet 17 weeks after cutting of Diploid (D) and tetraploid (T) numbers of *Escallonia rosea* (Diploid = grey, Tetraploid = white).

**Supplementary Figure 3**: The length of the new apical shoot (NSL) and its internode length (NSIL) of the 5 week old rooted cuttings. The length of the branches (BL), their internode lengths (BIL) , and the axillary budburst (%) of the pinched plantlet 17 weeks after cutting of Diploid (D) and tetraploid (T) numbers of *Escallonia rubra* (Diploid = grey, Tetraploid = white).

**Supplementary Figure 4**: Analysis of *Escallonia rosea* diploid (D) and tetraploid (T) numbers. Top view was analyzed for plant area (cm²), the circularity, and the % of the area of the minimal bounding circle (MBC) filled with the plant. Side view was analyzed for plant area (cm²), plant width (cm) and height (cm), and the % of the area of the bounding rectangle (BR) filled with the plant (Diploid = grey, Tetraploid = white)*.*

**Supplementary Figure 5**: Analysis of *Escallonia rubra* diploid (D) and tetraploid (T) numbers. Top view was analyzed for plant area (cm²), the circularity, and the % of the area of the minimal bounding circle (MBC) filled with the plant. Side view was analyzed for plant area (cm²), plant width (cm) and height (cm), and the % of the area of the bounding rectangle (BR) filled with the plant (Diploid = grey, Tetraploid = white)*.*
